# Cost-effectiveness analysis of acupuncture compared with usual care for acute non-specific low back pain: secondary analysis of a randomised controlled trial

**DOI:** 10.1177/09645284211055747

**Published:** 2021-11-30

**Authors:** Trygve Skonnord, Arne Fetveit, Holgeir Skjeie, Mette Brekke, Margreth Grotle, Atle Klovning, Eline Aas

**Affiliations:** 1 Department of General Practice, Institute of Health and Society, University of Oslo, Oslo, Norway; 2 General Practice Research Unit (AFE), Department of General Practice, Institute of Health and Society, University of Oslo, Oslo, Norway; 3 Department of Physiotherapy, Faculty of Health Sciences, Oslo Metropolitan University, Oslo, Norway; 4 Research and Communication Unit for Musculoskeletal Health (FORMI), Oslo University Hospital, Oslo, Norway; 5 Department of Health Management and Health Economics, Institute of Health and Society, University of Oslo, Oslo, Norway; 6 Health Services Research Unit, Akershus University Hospital, Lørenskog, Norway

**Keywords:** acupuncture, cost-effectiveness, low back pain, randomised controlled trial

## Abstract

**Objective:**

To assess the cost-effectiveness of a single treatment session of acupuncture, when applied in addition to usual care for acute low back pain (ALBP).

**Methods:**

Secondary analysis of a multicentre randomised controlled trial in Norwegian general practice. In total, 171 participants with ALBP ⩽14 days were randomised to a control group (CG) receiving usual care or to an acupuncture group (AG) receiving one additional session of Western medical acupuncture alongside usual care. Primary outcome measures for this cost-effectiveness analysis were quality-adjusted life years (QALYs), health care costs and societal costs at days 28 and 365, the incremental cost-effectiveness ratio (ICER) and net monetary benefit (NMB). The NMB was calculated on the basis of the Norwegian cost-effectiveness threshold of NOK 275,000 (USD 35,628) per QALY gained. Missing data were replaced by multiple chained imputation.

**Results:**

Eighty-six participants in the CG and 81 in the AG were included in the analysis. We found no QALY gain at day 28. At day 365, the incremental QALY of 0.035 was statistically significant. The differences in health care costs and societal costs were not statistically significant. Three out of four calculations led to negative ICERs (cost saving) and positive NMBs. For the health care perspective at day 365, the ICER was USD –568 per QALY and the NMB was USD 1265, with 95.9% probability of acupuncture being cost-effective.

**Conclusion:**

To our knowledge, this is the first cost-effectiveness analysis of acupuncture for ALBP. The findings indicate that acupuncture may be cost-effective from a 1-year perspective, but more studies are needed.

**Trial registration number:**

NCT01439412 (ClinicalTrials.gov).

## Introduction

Globally, low back pain (LBP) is a major cause of disability.^1,2^ Most episodes of LBP are classified as non-specific, and most affected patients recover within 1 month.^1,3^ However, as LBP is very common and many people experience recurrences or develop chronic pain, the burden for each patient and the costs for society are significant.^1,2^ These costs can be reported as health care costs and societal costs, due to absence from work or loss of productivity.

The treatment of acute low back pain (ALBP) usually takes place in primary health care and consists of information and education to avoid bed rest and to stay active.^4^ Recent guidelines have focused less on pharmacological care^5–7^ and, as in the American College of Physicians (ACP) guideline, more on non-pharmacological treatments before resorting to medication.^5^ Acupuncture is one of the non-pharmacological treatments mentioned in the ACP guideline for both chronic and ALBP. For chronic LBP, acupuncture has been shown to reduce pain and improve function in the short term compared with no treatment.^8–10^ However, there is insufficient evidence for the use of acupuncture in ALBP.^8,9^ Recently, we published the results of a multicentre randomised controlled trial (RCT) studying the effectiveness of adding acupuncture to usual care for ALBP.^11^ We did not find a statistically significant difference between the group receiving acupuncture and usual care (AG), and the control group (CG) that only received usual care, in measures of time to recovery (primary outcome), disability, absence from work and health-related quality of life (HRQoL). The small, statistically significant differences in the secondary outcomes of pain, global improvement and medication were not considered clinically relevant.^11^

There is a need for pragmatic studies evaluating the cost-effectiveness of acupuncture added to usual care, compared with usual care alone.^12,13^ In a systematic review by Andronis et al.,^12^ acupuncture for chronic LBP was found to be likely cost-effective; however, to our knowledge, no previous trials of acupuncture for ALBP have evaluated the cost-effectiveness of the treatment. One RCT reported by Nicolian et al.^14^ found acupuncture and usual care to be less costly and more effective than usual care alone in the treatment of pelvic pain and LBP in pregnancy.

The present study aimed to evaluate whether a single treatment session of acupuncture for ALBP, when applied in addition to usual care, was cost-effective compared with usual care alone.

## Methods

### Study design, material and treatment

This cost-effectiveness analysis was embedded in the Acuback study,^11,15^ a multicentre RCT with a 1-year follow-up. The details of the study design, sample size calculation, recruitment, randomisation, blinding and data collection in the Acuback study are given in the study protocol^15^ and in the paper reporting the clinical results.^11^ Briefly, the trial was conducted in 11 general practitioner (GP) clinics in Norway between March 2014 and March 2017. The study used a parallel design for the acupuncture group (AG) and the CG in an allocation ratio of 1:1, using a web-based randomisation system developed and administered by the Unit of Applied Clinical Research, Norwegian University of Science and Technology (NTNU),^16^ with various block sizes. The study needed to include 270 participants for the primary outcome, according to the sample size calculation.^15^

Participants in the trial were adults aged 20–55 years contacting their GP office with non-specific ALBP of 14 days duration or less, who gave informed consent. Patients with nerve root affection, so-called ‘red flags’ (risk factors for serious disease), pregnancy, disability pension, sick leave of more than 14 days and those who had received acupuncture treatment during the last month were excluded. All data were collected in electronic surveys at 19 different time points, one before and one after treatment on day 0, then daily for the first 2 weeks, then after 4 weeks, 12 weeks and 1 year. SESAMe software was used to manage the logistics of the surveys.^17^

The CG received treatment according to the Norwegian national guidelines,^18^ consisting of activity advice, analgesic medication (paracetamol and/or ibuprofen) and any eventual sick leave. The AG received one session of Western medical acupuncture treatment in addition to the same usual care as the CG, as described in the published protocol.^15^

The trial was prospectively registered at ClinicalTrials.gov (NCT01439412) on 23 September 2011, prior to recruitment of the first participant in March 2014. Ethical approval was given by the Regional Ethics Committee of South-Eastern Norway (reference 2013/611/REK sør-øst A). The reporting of the study follows the CONSORT statement^19^ and the STRICTA recommendations.^20^

### Treatment effect and utilities

To estimate HRQoL, we used the EuroQol 5-dimensions 3-level (EQ-5D-3L) utility index.^21^ For each of the five dimensions (mobility, self-care, daily activities, pain/discomfort and anxiety/depression), the patients reported no problem, some problems or severe problems. The EQ-5D-3L was collected at baseline and 1, 2, 4, 12 weeks and 1 year after treatment. Using the UK tariff for time trade-off,^22^ the scores were used to calculate the quality-adjusted life years (QALYs) at day 28 and day 365, which express the health gains in the cost-effectiveness analysis.

The cost-effectiveness threshold (willingness to pay, WTP) for LBP was based on the Norwegian governmental report no. 34 to the parliament with a value of NOK 275,000 (USD 35,628) per QALY.^23^ This threshold is valid just for the health care perspective.

### Health care and societal costs

For the cost-effectiveness analysis, we estimated both health care and societal costs at day 28 and day 365. The health care costs included direct costs for the study treatment (one consultation with the GP), reported use of medication (from every time point), estimation of extra consultations with the GP based on reports from every time point of work absence, and use of medication. In addition, day 365 also included costs of other therapies such as physiotherapy, chiropractic, osteopathy, naprapathy, acupuncture and surgery, estimated by reported types of therapy and number of new LBP episodes.

In Norway, 59% of GPs are specialists in family medicine with higher charges per consultation than non-specialist GPs. Therefore, GP charges were weighted according to this variation.^24^ Moreover, the GP costs were adjusted for per capita subsidy and differentiated by consultation time (⩽20 or >20 min). To calculate the unit costs of other therapies, surgery and medication, we used information from official websites, such as those from the Norwegian Physiotherapist Association and other therapists (Table 1). Because we did not have the necessary data for calculation of transport costs related to the various treatments, these costs were not included in the analyses. We used costs in NOK for 2018, converted to US dollars, where USD 1 = NOK 7.7186 (March 2018).

**Table 1.** Cost categories, units, valuation and unit price.

Cost categories	Unit	Valuation	Unit price	
			USD	NOK
General practitioner (GP)	Per treatment	Charge^a^	58	450
	Per phone prescription	Charge	14	110
Physiotherapist	Per treatment	Charge	73	560
Other therapists	First treatment	Charge	97	750
	Later treatments	Charge	58	450
Back surgery (day surgery)	Per surgery	Charge	6,024	46,500
Acupuncture equipment	Per treatment	Cost	13	100
Non-opioid medication	Per defined daily doses	Cost^b^	0,5	3.9
Opioid medication	Per defined daily doses	Cost^b^	1,7	13.2
Productivity loss (away from work)	Per day	Wage rate^c^	319	2,463

All numbers in US dollars (USD) and Norwegian krone (NOK) for March 2018.

a GP charge: mean, calculations used different charges for ⩽20 min and >20 min.

b Medication cost: estimated price weighted by different medication types and packages.

c Wage rate: mean, calculations used differentiated salaries by sex and age in Norway.

Because we did not have exact data for the number of GP visits or other therapies (physiotherapy, chiropractic, osteopathy, naprapathy, acupuncture) for the estimation of costs, we used the following assumptions of moderate use of health care services: one consultation with the GP for one new episode of ALBP; two consultations for two episodes; three consultations for three to four episodes; and four consultations for five or more new episodes. For the other therapies, we assumed that they comprised four treatments per new episode. We also performed sensitivity analysis with a lower and a higher use of health care services, using values of approximately 50% and 150% of the moderate estimation, respectively. In addition, we performed a sensitivity analysis where one participant who underwent surgery was excluded.

The reported use of medication was converted to the defined daily dose (DDD) of non-opioid and opioid medication. The costs were calculated from prices from the Norwegian Medicines Agency^25^ and Pharmacy Selling Prices,^26^ including value-added tax (VAT).

Costs for absence from work were based on official statistics of average wages by sex and age groups,^27^ adjusted for the proportion of part-time positions (official statistics) and the reported percentage of sick leave at each time point. In addition, wages included social costs of 40%. The mean wage rate is given in Table 1. For those without work absence, the societal costs were equal to the health care costs.

### Missing data

Missing data are a common problem in RCTs.^28^ In addition to the aforementioned estimates for the use of health care services, we used the following assumptions. Work absence was estimated from self-reported absence from work at each time point, combined with reported sick leave for the past year at day 365. When missing data of work absence occurred after valid reporting, we used the last known value. When the patients did not report use of medication, we used the value zero.

Missing data for total costs at days 28 and 365 were solved using multiple chained imputation. Missing data for HRQoL were imputed in three steps. First, we performed a mean imputation of missing baseline values. Then, for missing values between two observation points, we assumed the mean value of the two observed values. Finally, a multiple chained imputation was performed together with the imputation of the costs.

There is a probability that the missing data are not random if, for example, the participants stop reporting after recovering from the condition. Then, multiple chained imputations are preferable; on the contrary, adding more variables with missing data makes it difficult to achieve a stable imputation model.^28^ Therefore, we performed several sensitivity analyses to test the robustness of the analysis, with the following scenarios: no imputation, manual imputation of both HRQoL and costs, both with and without the participant that underwent surgery. Finally, we performed combinations of multiple chained imputations of HRQoL and different variations of manual imputations of the costs (no imputation, mean by group and mean of all participants).

### Statistical analysis

The statistical analyses in this cost-effectiveness analysis were performed using the programme StataSE V.16.1. As described in the clinical results,^11^ we present per-protocol analysis instead of intention-to-treat analysis, due to trial logistic reasons.

Cost-effectiveness was estimated by the incremental cost-effectiveness ratio (ICER), as defined by the incremental costs (the difference in costs between the AG and the CG) relative to QALYs gained as

$$ICER=\frac{Costs_{AG}-Costs_{CG}}{QALY_{AG}-QALY_{CG}}$$

To find the QALYs gained, the trapezoidal method was used to estimate the area under the curve by combining utility indexes and time.^29^ To avoid ambiguous interpretation of the ICER, the net monetary benefit (NMB) as defined by incremental QALYs multiplied by the threshold minus the incremental costs was calculated as

$$\begin{array}{l} NMB=\left(\left(QALY_{AG}-QALY_{CG}\right)*WTP\right)\\ \;\;\;\;\;\;\;\;\;\;\;\;\;\;\;-(Costs_{AG}-Costs_{CG}) \end{array}$$

If the NMB was equal to or greater than zero, acupuncture would be considered cost-effective. Uncertainty was analysed by the non-parametric bootstrap method with 1000 iterations.^30^ The results are presented in scatter plots, where the incremental effect of each analysis is plotted on the x-axis and the incremental cost on the y-axis.^29^ As the WTP threshold is valid for the health care perspective, these plots also contain an axis representing the threshold of NOK 275,000 (USD 35,268) per QALY gained.

## Results

A total of 185 participants were randomised into the two groups, of which 167 were included in the analysis: 86 in the CG and 81 in the AG as shown in the study flow chart published previously (Supplemental file 1).^11^ This number was less than the planned sample size, even though the period of inclusion was extended by 1 year with the last follow-up in March 2018. The response rate of the included participants decreased over time to 76.0% after 1 year, with an overall average response rate of 87.4%. There were no statistically significant differences between the groups in response rate.

Table 2 presents the baseline variables of socio-demographic data and clinical features of the participants. There were no statistically significant differences between the groups in any of the variables. The two groups did not differ statistically in work absence at any of the time points (Supplemental file 2). One specific difference between the groups was that one participant in the AG underwent an operation for sciatica during the study period.

**Table 2.** Baseline characteristics of participants in the two treatment groups (n = 167).

Characteristic	Control (n = 86)	Acupuncture (n = 81)
Age (years), mean (95% CI)	39.3 (37.3–41.3)	39.8 (37.3–42.4)
Female, n (%)	44 (51.2)	41 (50.6)
Born in Norway, n (%)	78 (92.9)	69 (88.5)
Level of education > 13 years, n (%)	28 (33.3)	30 (38.5)
Work status		
Employed, n (%)	77 (91.7)	70 (87.5)
Student, n (%)	7 (8.3)	6 (7.5)
Unpaid work, n (%)	1 (1.2)	1 (1.3)
Unemployed, n (%)	2 (2.4)	3 (3.8)
Sick leave, n (%)	3 (3.6)	3 (3.8)
BMI		
<25 (normal), n (%)	28 (33.3)	30 (38.5)
25.0–29.9 (overweight), n (%)	29 (34.5)	29 (37.2)
>30 (obese), n (%)	27 (32.1)	19 (24.4)
Smoking, n (%)	20 (23.8)	14 (17.9)
Previous LBP, n (%)	63 (73.3)	58 (71.6)
Back pain intensity (0–10), mean (95% CI)	6.3 (5.9–6.7)	6.2 (5.7–6.6)
RMDQ (0–24), mean (95% CI)	14.8 (13.8–15.7)	15.0 (14.1–15.9)
EQ-5D-3L, mean (95% CI)	0.40 (0.33–0.48)	0.41 (0.34–0.48)
DDD non-opioid medication, mean (95% CI)	0.66 (0.48–0.85)	0.93 (0.71–1.15)
DDD opioid medication, mean (95% CI)	0.09 (0.03–0.15)	0.09 (0.02–0.16)
SHC, mean (95% CI)	11.25 (9.64–12.86)	9.12 (7.90–10.33)
Missing	2	3

CI: confidence interval; BMI: body mass index; LBP: low back pain; RMDQ: Roland Morris disability questionnaire, a higher score represents greater overall disability; DDD: defined daily dose; SHC: subjective health complaints, a higher score means more reported health complaints; EQ-5D-3L: EuroQol 5-dimension 3-level utility index, a higher score represents better health state.

Data are n (%) or mean (95% CI). There were no significant differences between the groups in any of the variables.

The observed results of the cost-effectiveness analysis of the observed data are given in Table 3, while the imputed and bootstrapped results are presented in Table 4, along with the sensitivity analyses.

**Table 3.** Observed results of costs (USD) and utilities (QALYs) with subsequent incremental cost-effectiveness ratio (ICER) and net monetary benefit (NMB) at different time points.

Treatment group	Cost (USD) mean (95% CI)	△ cost	QALYs mean (95% CI)	△ QALYs	ICER^a^ (USD/QALY)	NMB^b^ (USD)
Health care perspective day 28						
CG (n = 59)	89 (77–102)		0.05618 (0.05291–0.05945)			
AG (n = 56)	99 (85–113)	10	0.05674 (0.05412–0.05937)	0.00056	17,857	10
Health care perspective day 365						
CG (n = 52)	645 (401–890)		0.8049 (0.7639–0.8459)			
AG (n = 54)	648 (266–1029)	3	0.8536 (0.8318–0.8754)	0.0487	62	1732
Societal perspective day 28						
CG (n = 51)	2495 (1625–3,365)		0.05618 (0.05291–0.05945)			
AG (n = 53)	1904 (1126–2683)	−591	0.05674 (0.05412–0.05937)	0.00056	−1,055,357	611
Societal perspective day 365						
CG (n = 44)	10,343 (3403–17,283)		0.8049 (0.7639–0.8459)			
AG (n = 51)	5869 (2639–9100)	−4474	0.8536 (0.8318–0.8754)	0.0487	−91,887	6209

△: incremental (difference); AG: acupuncture group; CG: control group; QALY: quality-adjusted life year; CI: confidence interval; USD: United States dollar; WTP: willingness to pay (threshold value).

a Incremental cost-effectiveness ratio (ICER) = (costs AG − costs CG)/(QALY AG − QALY CG).

b Net monetary benefit (NMB) = ((QALY AG − QALY CG) × WTP) − (costs AG − costs CG).

**Table 4.** Bootstrapped results and sensitivity analysis, showing differences in mean costs (USD), incremental costs, utilities (QALYs) and incremental QALYs with subsequent incremental cost-effectiveness ratio (ICER) and net monetary benefit (NMB).

Sensitivity analysis	Cost (USD)	△ cost	QALYs	△ QALYs	ICER^a^	NMB^b^
Treatment group	Mean (95% CI)		Mean (95% CI)		(USD/QALY)	(USD)
Health care perspective day 28, MI HRQoL + costs						
CG (n = 86)	87 (87–88)		0.05620 (0.05611–0.05629)			
AG (n = 81)	96 (95–96)	8	0.05623 (0.05615–0.05631)	0.00003	266,667	−7
Health care perspective day 365, MI HRQoL + costs						
CG (n = 86)	560 (554–566)		0.8199 (0.8189–0.8209)			
AG (n = 81)	540 (532–549)	−20	0.8549 (0.8543–0.8554)	0.0350	−572	1266
Societal perspective day 28, MI HRQoL + costs						
CG (n = 86)	2346 (2324–2368)		0.05620 (0.05611–0.05629)			
AG (n = 81)	1854 (1831–1876)	−492	0.05623 (0.05615–0.05631)	0.00003	−16,400,000	493
Societal perspective day 365, MI HRQoL + costs						
CG (n = 86)	5941 (5825–6058)		0.8199 (0.8189–0.8209)			
AG (n = 81)	5404 (5303–5504)	−538	0.8549 (0.8543–0.8554)	0.0350	−15,389	1784
Societal perspective day 365, MI HRQoL + costs, low use of health care services						
CG (n = 86)	5804 (5688–5920)		0.8199 (0.8189–0.8209)			
AG (n = 81)	5286 (5185–5386)	−519	0.8549 (0.8543–0.8554)	0.0350	−14,846	1765
Societal perspective day 365, MI HRQoL + costs, high use of health care services						
CG (n = 86)	6112 (5995–6229)		0.8199 (0.8189–0.8209)			
AG (n = 81)	5532 (5431–5633)	−580	0.8549 (0.8543–0.8554)	0.0350	−16,590	1826
Societal perspective day 365, before imputation						
CG (n = 44)	10,947 (3772–18,121)		0.8031 (0.7614–0.8447)			
AG (n = 51)	5983 (2784–9182)	−4964	0.8504 (0.8270–0.8738)	0.0473	−104,903	6650
Societal perspective day 365, manual imputation HRQoL + costs						
CG (n = 86)	9145 (5740–12,551)		0.8176 (0.7902–0.8451)			
AG (n = 81)	7796 (4523–11,069)	−1349	0.8501 (0.8334–0.8667)	0.0324	−41,623	2504
Societal perspective day 365, manual imputation HRQoL + costs, excluded surgery						
CG (n = 86)	8932 (5427–12,436)		0.8181 (0.7901–0.8462)			
AG (n = 80)	6958 (3965–9951)	−1974	0.8553 (0.8425–0.8682)	0.0372	−53,079	3299
Societal perspective day 365, MI HRQoL, no imputation costs						
CG (n = 86)	9481 (9295–9667)		0.8204 (0.8195–0.8214)			
AG (n = 81)	7334 (7193–7476)	−2147	0.8544 (0.8539–0.8550)	0.0340	−63,203	3357
Societal perspective day 365, MI HRQoL, manual imputation costs (mean all)						
CG (n = 86)	9069 (8962–9177)		0.8204 (0.8195–0.8214)			
AG (n = 81)	7659 (7558–7759)	−1410	0.8544 (0.8539–0.8550)	0.0340	−41,507	2620
Societal perspective day 365, MI HRQoL, manual imputation costs (mean by group)						
CG (n = 86)	9587 (9475–9698)		0.8204 (0.8195–0.8214)			
AG (n = 81)	7482 (7381–7584)	−2105	0.8544 (0.8539–0.8550)	0.0340	−61,966	3315

△: incremental (difference); AG: acupuncture group; CG: control group; QALY: quality-adjusted life year; CI: confidence interval; USD: United States dollar; WTP: willingness to pay (threshold value); MI: multiple imputation; HRQoL: health-related quality of life.

a Incremental cost-effectiveness ratio (ICER) = (costs AG − costs CG)/(QALY AG − QALY CG).

b Net monetary benefit (NMB) = ((QALY AG − QALY CG) × WTP) − (costs AG − costs CG).

The main differences from the observed to the bootstrapped data were primarily lower incremental costs from a societal perspective at day 365, lower incremental QALYs at day 28 and lower standard deviations (SDs). On the basis of the large SDs for the observed data and the recommended methods for handling missing data,^28^ we have presented the imputed and bootstrapped results as our main results. The mean health care costs at day 28 were USD 96 (SD 6) in the AG and USD 87 (SD 5) in the CG, and at 1-year follow-up USD 540 (SD 137) in the AG and USD 560 (SD 93) in the CG. Societal costs, including absence from work, were estimated to be USD 1854 (SD 360) for the AG and USD 2346 (SD 352) for the CG at day 28, and after 1 year, USD 5404 (SD 1619) in the AG and USD 5941 (SD 1876) in the CG.

HRQoL measured by EQ-5D-3L did not show significant differences at any time point (Supplemental file 3). After the imputation process with calculation of QALYs was conducted, the observed difference between the groups at day 28 was 0.00003 QALYs (95% confidence interval (CI), –0.00008 to 0.00014), and at day 365 the difference was 0.0350 (95% CI, 0.0338 to 0.0361).

From a health care perspective, the ICERs at days 28 and 365 were USD 266,667 and USD –572 per QALY gained, respectively, while from a societal perspective, the ICERs at days 28 and 365 were USD –16,400,000 and USD –15,389 per QALY gained, respectively.

The NMB was positive in three out of four calculations. With regard to health care costs at days 28 and 365, the NMB values were USD –7 and USD 1266, respectively; for societal costs, the values were USD 493 and USD 1784, respectively.

We performed several sensitivity analyses, and the results of costs and utilities from a societal perspective at day 365 for different scenarios are given in Table 4. In principle, all the analyses reflect the main results, with negative incremental costs, positive incremental QALYs, negative ICERs and positive NMBs, but with different values, depending on the method used.

**Figure 1.**
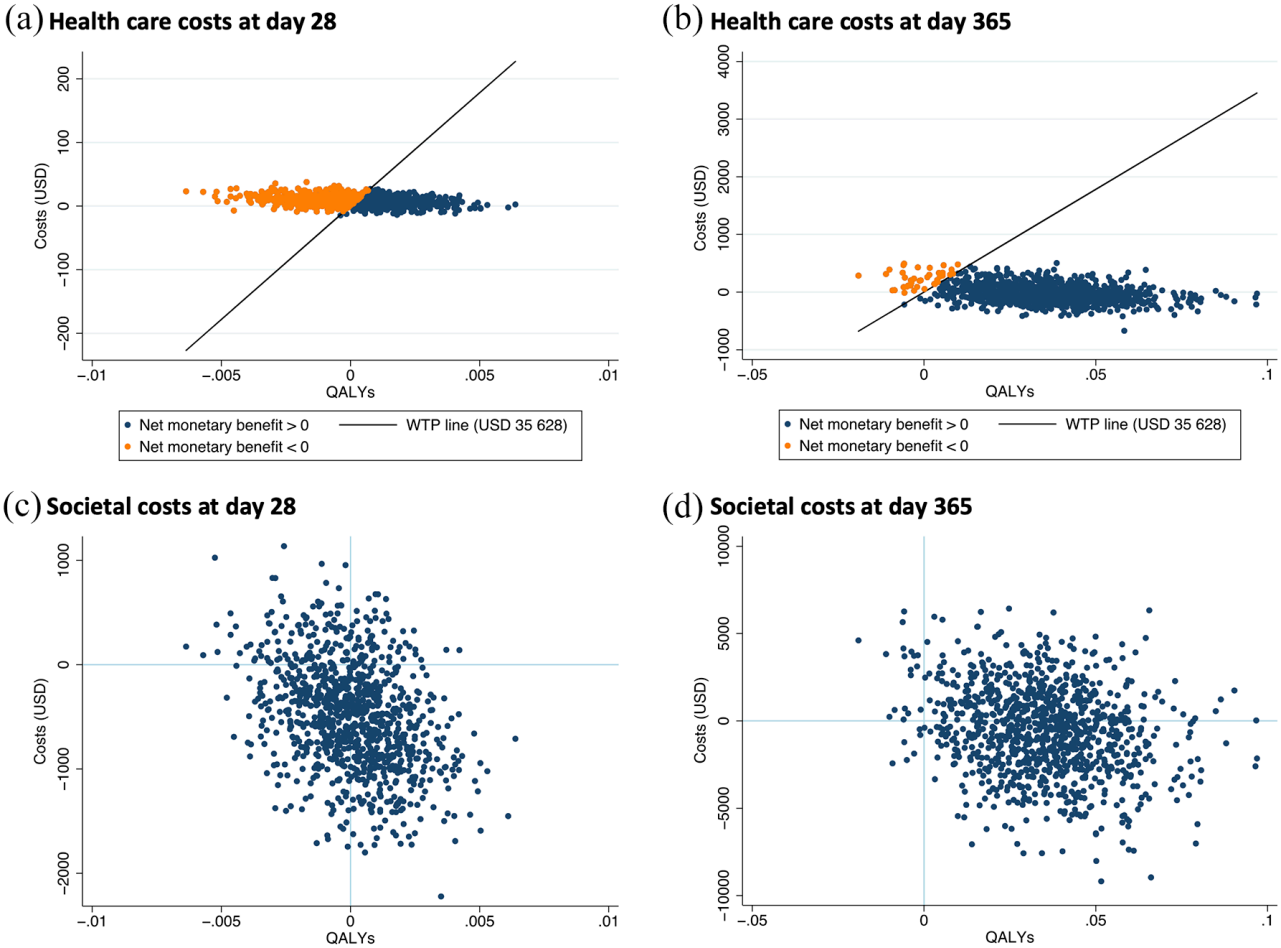
Scatter plot of incremental health care costs and incremental quality-adjusted life years (QALYs) at day 28 (a) and day 365 (b); and incremental costs from a societal perspective and incremental QALYs at day 28 (c) and day 365 (d).

The uncertainty analysis of both health care costs and societal costs at day 28 and 1 year is shown in Figure 1. The ICERs were estimated with the assumption of a moderate use of health care services, and sensitivity analysis with low or high use of health care services did not change the results substantially. From the bootstrapped results, the majority of the replicated dataset indicated that acupuncture was cost-saving and provided a QALY gain at day 365. At day 28, the incremental QALY was zero for both health care costs and societal costs. Given the threshold cost of NOK 275,000, the probability of acupuncture being cost-effective according to health care costs at days 28 and 365 was 46.1% and 95.9%, respectively; for societal costs, the probability of acupuncture being cost-effective was 81.5% and 74.1%, respectively.

## Discussion

To our knowledge, this is the first published trial analysing cost-effectiveness of acupuncture for ALBP. At day 28, there was no difference in QALYs between the groups, but after 1-year follow-up, we found a statistically significant QALY gain for the acupuncture group. The present differences in costs at days 28 and 365 were not statistically significant, from either a health care perspective or a societal perspective. At day 365, we found a probability of 95.9% of acupuncture being cost-effective from a health care perspective and 74.1% from a societal perspective.

Researchers have called for further studies exploring the cost-effectiveness of acupuncture and other treatments of LBP.^4,31^ This is the first study to report the cost-effectiveness of acupuncture treatment provided to patients with an episode of ALBP seeking primary care. A strength of the study is the perceived small difference in attention bias between the groups due to the standardised intervention procedures.^11^

The main limitation of the study is the significant amount of missing data for calculation of both the QALYs and the costs. Thus, we included both results with different assumptions regarding imputation. A further challenge involves the sparse data collected between days 28 and 365, with data from only day 84 in this period. These two limitations explain why we conducted imputation on total costs on days 28 and 365 (instead of on details of health care utilisation), which Faria et al.^28^ note is an appropriate method when the missing data of the different parts of the costs have the same pattern. This is to achieve a stable imputation model, which is challenging when adding more variables with missing data.

The inclusion rates were lower than expected, which led to inadequate power. As a consequence, our results have wide CIs, and the estimated effectiveness lacks precision. The low power and wide CIs increase the probability of spurious findings. Another limitation is that we did not include a health economist in the project group in the early phases of the trial, which could have resulted in more detailed data collection on costs.

Our trial was performed in Norwegian general practice. The external validity may thus be limited by different costs for both health care services and societal costs in other settings and other countries. The use of just one short treatment session of acupuncture was based on clinical experience, where GPs frequently experienced faster recovery even after the first treatment.^11,15^ This is a less comprehensive treatment strategy than usual,^32^ but was chosen after a comprehensive feedback evaluation to reduce the difference in attention bias between the groups. More treatment sessions of longer duration would have affected the health care costs, but could also have affected the costs and QALYs, and the total difference from our present results cannot be predicted.

It is very relevant to discuss whether a single treatment session of acupuncture on a self-limited condition such as ALBP can cause a difference in QALY after 1 year, especially when there was no difference after 4 weeks. The present study was a pragmatic, non-blinded trial, and a persisting effect of positive expectations might exist; most participants preferred to be in the AG and believed, prior to the treatment,^11^ that acupuncture would help them as it has been shown to have an effect in pain studies.^33^

Our findings from the cost-effectiveness analysis add to the main results in the Acuback trial (i.e. no significant finding on the number of days to recovery, or pain and global improvement),^11^ indicating that acupuncture is a cost-effective strategy (cost per QALY gained). Although the analysis of EQ-5D-3L at each observation point (Supplemental file 3) did not show statistically significant differences, after imputing and bootstrapping these data, we found a QALY gain for day 365, which is the driving mechanism for the positive NMBs. The trapezoidal method might have contributed to increase the small differences in EQ-5D-3L through the calculation of QALYs, estimating the area under the curve by combining utility indices and time.

Health economic analysis depends on differences both in costs and health outcomes (i.e. QALYs). Hence, the results could favour one alternative over another even though there are no significant differences in health outcomes, if there are differences in costs. The difference in costs between the groups was mainly driven by productivity gain, even if the difference in work absence was not statistically significant. The cost-effectiveness analysis combines several variables for each individual, which accords with the systematic review of Lin et al.^34^ of cost-effectiveness of general practice care for LBP.

The sensitivity analyses showed that the multiple chained imputation process led to lower incremental costs than other imputation models and analyses without imputation of missing data. In the process of imputation and bootstrapping, the extreme observations will count less, and the values will be drawn both to the mean and to lower values. This can occur if more recovered participants have missing data that are imputed.

We have chosen to present both health care costs and societal costs in this study. The WTP threshold in Norway of 275,000 NOK (USD 35,628) per QALY gained is based on a health care perspective.^23^ The Norwegian threshold is similar to the UK threshold of the National Institute for Health and Care Excellence (NICE) (GBP 20,000–30,000).^35^ A working group on behalf of the Norwegian Ministry of Health and Care Services^36^ has suggested a higher threshold for more severe conditions, with the highest WTP threshold for loss of health of more than 20 years of 825,000 NOK (106,885 USD) per QALY gained. Although LBP is not a serious condition with high morbidity, it is nevertheless common and can cause many years of living with disability, productivity loss and reduced quality of life.^2^ The threshold of 275,000 NOK is used in other Norwegian LBP studies,^37,38^ but there is a possibility that considering just the health care costs could lead to lower priority of this group of patients, as the loss of productivity contributed to most of the societal costs both in earlier studies and in our study.

Lin et al.^34^ noted that GP care was associated with low health care costs, but with higher societal costs than other treatments; thus, most treatments were found to be cost-effective compared with GP care. Acupuncture was shown to be cost-effective in a study of chronic LBP by Ratcliffe et al.,^39^ and our findings on cost-effectiveness for ALBP in the longer term are similar to both that study as well as to trials of chronic LBP reported by Andronis et al.^12^ One trial of the use of acupuncture for pelvic pain and LBP in pregnancy also showed that acupuncture is cost-effective, resulting in lower societal costs and better health outcomes;^14^ however, in that case, the effect was measured in days with pain numeric rating scale (NRS) ⩽ 4, and not in QALYs, as in our study.

The present work adds new knowledge about the cost-effectiveness of acupuncture for ALBP as it is, to our knowledge, the only trial with this outcome. One possible clinical implication of the cost-effectiveness of acupuncture for ALBP is greater support for acupuncture as one of the non-pharmacological interventions as first-line treatment, as recommended in the ACP guidelines.^5^ The US Centers for Medicare & Medicaid Services (CMS.gov)^40^ began to cover acupuncture for chronic LBP in January 2020. Future cost-effectiveness analyses are clearly needed to provide more evidence on the costs and health outcomes of using acupuncture for ALBP.

## Conclusion

In this study, we added one acupuncture treatment to usual care for patients with ALBP compared with usual care alone. The cost-effectiveness analysis indicated that acupuncture was cost-effective from a health care perspective after 1 year. However, the results should be interpreted with caution, as this is (to our knowledge) the first trial presenting cost-effectiveness analysis for acupuncture for ALBP. Some limitations of the present trial should also be borne in mind, including low power and inconclusive effectiveness in the main trial. Thus, further research is needed to confirm if acupuncture is a cost-effective treatment for ALBP.

## Supplemental Material

sj-pdf-1-aim-10.1177_09645284211055747 – Supplemental material for Cost-effectiveness analysis of acupuncture compared with usual care for acute non-specific low back pain: secondary analysis of a randomised controlled trial Click here for additional data file. Supplemental material, sj-pdf-1-aim-10.1177_09645284211055747 for Cost-effectiveness analysis of acupuncture compared with usual care for acute non-specific low back pain: secondary analysis of a randomised controlled trial by Trygve Skonnord, Arne Fetveit, Holgeir Skjeie, Mette Brekke, Margreth Grotle, Atle Klovning and Eline Aas in Acupuncture in Medicine

sj-tiff-1-aim-10.1177_09645284211055747 – Supplemental material for Cost-effectiveness analysis of acupuncture compared with usual care for acute non-specific low back pain: secondary analysis of a randomised controlled trial Click here for additional data file. Supplemental material, sj-tiff-1-aim-10.1177_09645284211055747 for Cost-effectiveness analysis of acupuncture compared with usual care for acute non-specific low back pain: secondary analysis of a randomised controlled trial by Trygve Skonnord, Arne Fetveit, Holgeir Skjeie, Mette Brekke, Margreth Grotle, Atle Klovning and Eline Aas in Acupuncture in Medicine

sj-tiff-2-aim-10.1177_09645284211055747 – Supplemental material for Cost-effectiveness analysis of acupuncture compared with usual care for acute non-specific low back pain: secondary analysis of a randomised controlled trial Click here for additional data file. Supplemental material, sj-tiff-2-aim-10.1177_09645284211055747 for Cost-effectiveness analysis of acupuncture compared with usual care for acute non-specific low back pain: secondary analysis of a randomised controlled trial by Trygve Skonnord, Arne Fetveit, Holgeir Skjeie, Mette Brekke, Margreth Grotle, Atle Klovning and Eline Aas in Acupuncture in Medicine
