# Supplementary material for: Cost-effectiveness analysis of acupuncture compared with usual care for acute non-specific low back pain: secondary analysis of a randomised controlled trial
Source: Acupunct Med. 2021 Nov 30;40(2):123–32. doi: 10.1177/09645284211055747 (PMC8873285; doi:10.1177/09645284211055747)
Supplement: sj-pdf-1-aim-10.1177_09645284211055747 – Supplemental material for Cost-effectiveness analysis of acupuncture compared with usual care for acute non-specific low back pain: secondary analysis of a randomised controlled trial [file sj-pdf-1-aim-10.1177_09645284211055747.pdf]

Supplemental file 2. Work absence and work presence in the treatment groups at the 1-year follow-up (n = 147).

	Baseline	Day 1	Day 2	Day 3	Day 4	Day 5	Day 6	Day 7	Day 8	Day 9	Day 10	Day 11	Day 12	Day 13	Day 14	Day 28	Day 84	Day 365
Control group (n=77)																		
Work absence (n)	3	38	42	39	35	35	28	23	21	21	19	18	16	16	15	7	3	6
Work absence (%)	4	49	55	51	45	45	36	30	27	27	25	23	21	21	19	9	4	8
Work presence (n)	74	29	27	28	32	31	40	44	45	45	46	43	47	46	46	49	53	47
Work presence (%)	96	38	35	36	42	40	52	57	58	58	60	56	61	60	60	64	69	61
Missing (n)	0	10	8	10	10	11	9	10	11	11	12	16	14	15	16	21	21	24
Acupuncture group (n=70)																		
Work absence (n)	3	31	32	30	28	28	25	17	16	14	15	14	11	11	11	8	9	9
Work absence (%)	4	44	46	43	40	40	36	24	23	20	21	20	16	16	16	11	13	13
Work presence (n)	67	36	36	38	38	37	41	48	46	46	47	47	50	47	51	53	51	51
Work presence (%)	96	51	51	54	54	53	59	69	66	66	67	67	71	67	73	76	73	73
Missing (n)	0	3	2	2	4	5	4	5	8	10	8	9	9	12	8	9	10	10
Total (n=147)																		
Work absence (n)	6	69	74	69	63	63	53	40	37	35	34	32	27	27	26	15	12	15
Work absence (%)	4	47	50	47	43	43	36	27	25	24	23	22	18	18	18	10	8	10
Work presence (n)	141	65	63	66	70	68	81	92	91	91	93	90	97	93	97	102	104	98
Work presence (%)	96	44	43	45	48	46	55	63	62	62	63	61	66	63	66	69	71	67
Missing (n)	0	13	10	12	14	16	13	15	19	21	20	25	23	27	24	30	31	34
