# Supplementary figures and images for: Cost-effectiveness analysis of acupuncture compared with usual care for acute non-specific low back pain: secondary analysis of a randomised controlled trial
Source: Acupunct Med. 2021 Nov 30;40(2):123–32. doi: 10.1177/09645284211055747 (PMC8873285; doi:10.1177/09645284211055747)

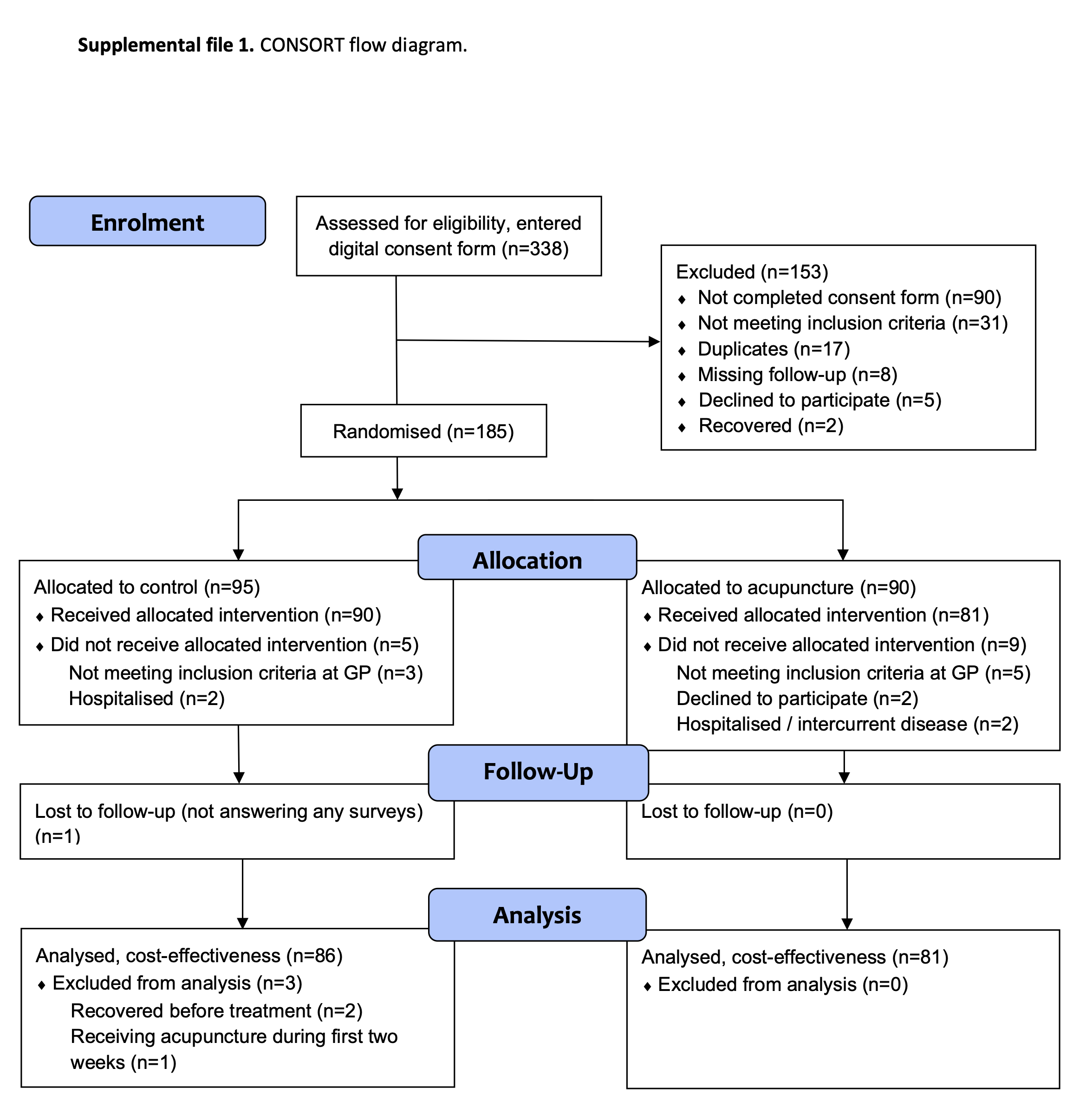

Supplement: sj-tiff-1-aim-10.1177_09645284211055747 – Supplemental material for Cost-effectiveness analysis of acupuncture compared with usual care for acute non-specific low back pain: secondary analysis of a randomised controlled trial [file sj-tiff-1-aim-10.1177_09645284211055747.tiff]

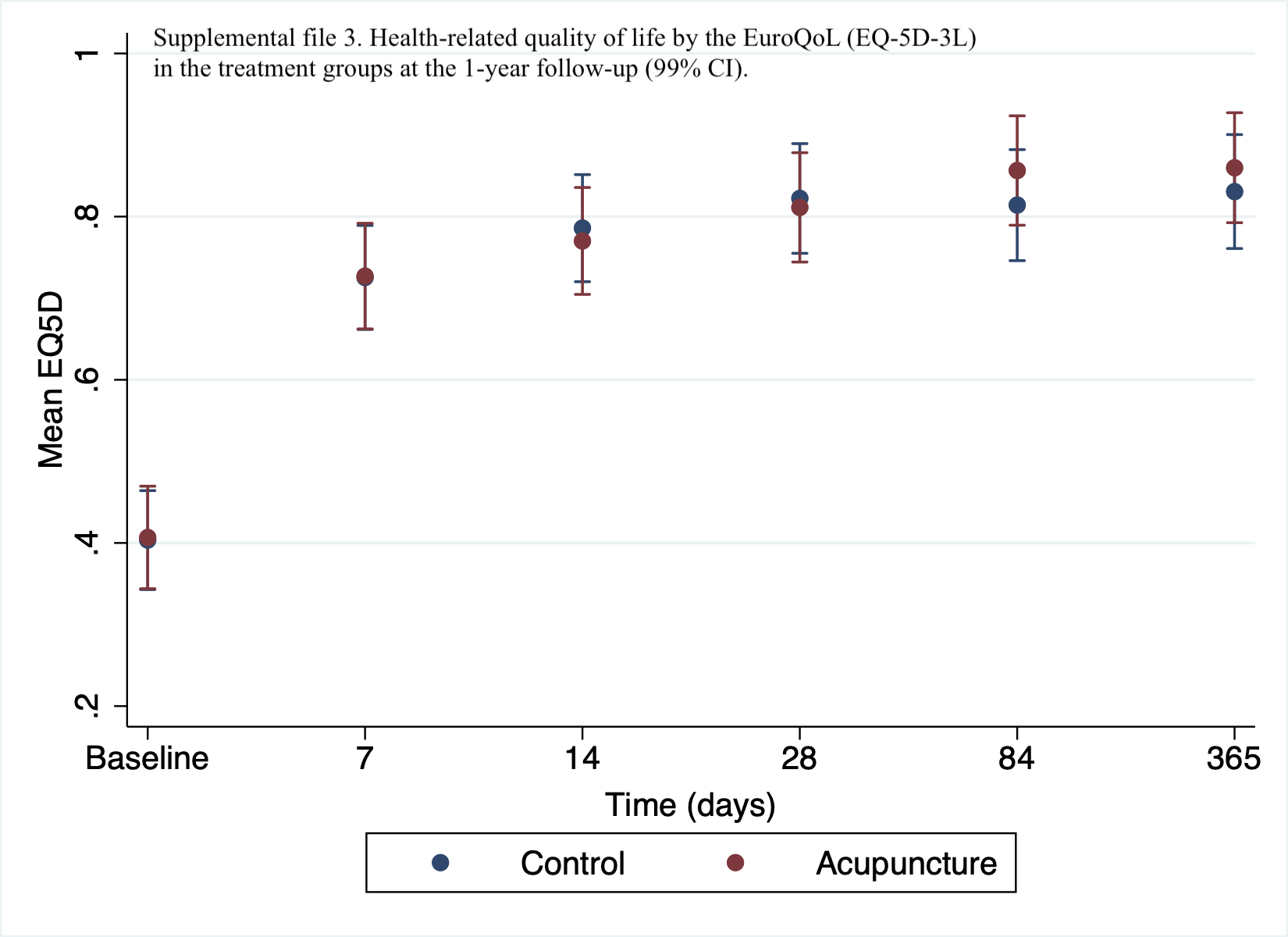

Supplement: sj-tiff-2-aim-10.1177_09645284211055747 – Supplemental material for Cost-effectiveness analysis of acupuncture compared with usual care for acute non-specific low back pain: secondary analysis of a randomised controlled trial [file sj-tiff-2-aim-10.1177_09645284211055747.tiff]
